# Determining information system end-user satisfaction and continuance intension with a unified modeling approach

**DOI:** 10.1038/s41598-024-57218-4

**Published:** 2024-03-22

**Authors:** Worku Mekonnen Tessema, Nadire Cavus

**Affiliations:** 1 Department of Computer Science, Debre Tabor University, Debra Tabor, Ethiopia; 2 Department of Computer Information Systems, Near East University, Nicosia, Cyprus; 3 Computer Information Systems Research and Technology Centre, Near East University, Nicosia, Cyprus

**Keywords:** Localization, System quality, Continuance intention, Integrated models, UTAUT, ECTM, ISS model, Energy science and technology, Engineering, Mathematics and computing

## Abstract

With the different characters of datatypes and large amount of data going to be managed in open-source database, localization to the specific linguistics is the major concern in Ethiopia, as the nation used different datatypes compared to the Gregorian systems. In this regard Amharic localization in open-source database can handle the difficulties in managing data for governmental and non-governmental organizations. Amharic Extension Module was introduced to governmental organizations for the data management capabilities. But, there is no research that can explore the system’s quality, the users’ satisfaction and intension of continuance of Amharic Extension Module from the perspective of both computer literates and illiterates. Therefore, this research work attempt or try to empirically examine and analyze the system quality, the users’ satisfaction and intension of continuance of Amharic Extension Module from the perspective of all users in POESSA The major purpose/aim of this study/research is to brand or make up the research break/gap in the area of localization specific to the Amharic locals, and to show the implication of the practical and theoretical way based on the results of the research. For this purpose, questionnaires were used for the collection of the research data. A total of 395 copies of the questionnaires were distributed and 385 of them are collected without any problem from the organization indicated herewith. The statistical analysis tools such as SPSS and AMOS, and methods such as Structural equation model were used for the analysis of the research data. The results of the research recommended and suggested that system quality can significantly influence confirmation. Meanwhile, confirmation can directly and significantly influence perceived usefulness, performance expectations, and satisfaction. Additionally, performance expectation, perceived usefulness and confirmation can significantly impact/influence satisfaction. The satisfaction directly and most importantly and significantly influences the continuance intension. Finally, the research delivers/provides a concert indication for the legitimacy and validity of the integrated and combined models of UTUAT, ECTM, and D&M ISS in the field of localizations which can be a hypothetical and theoretical foundation for Amharic Extension Module—AEM users’ and services of it.

## Introduction

Because of the enormously growing sizes of local language data that are a byproduct of current life, there is an increasing need for a suitable database that requires efficient and well-organized methods for the better management of data and for information retrieval. Storing, manipulation and accessing data in their original form is critical, because converted data can result in information loss^1^. For such data management cases, open source databases have evolved solely into the solutions in the organizations and enterprises.

Among the data management techniques used in open-source databases, localization is can be uses as the procedure of adapting a creation to novel linguistic and cultural content facts to a given locals. The Ethiopic script (also known as Amharic script/Ge'ez) is one of the most widely used writing methods for the Semitic language spoken in Ethiopia, and the linguistic comprises over 401 characters. Even though the Amharic language (Ge'ez) is now only used for liturgical purposes, the inscription is still widely used for the writing system and the official scripts of both Ethiopian and Eritrean Semitic languages such as Agew, Tigré and Amharic^2,3^. The Ethiopian ancient calendar differs from that of the Gregorian in the writing system of day, month, and year. Considering the Ethiopic calendar having 12 months, each with equal number of 30 days, and one more additional month (13th) comes at the end of the year with 5 or 6 days depending on whether this last year is a leap year^4,5^. As a consequence, a large volume of data in Ethiopic script uses the Ethiopian calendar and must be stored and manipulated in databases in accordance with the Ethiopian local system.

As community awareness of localization grows, there is a rising demand for massive amounts of data in databases and database services and facilities that is primarily imitated as aggressively demanding the monitoring of sensitive data/information from various sources in databases^6^. As data/information load increases, the conflict between increasing information request and shortage and unequal supply of information resources becomes a main impediment to the improvement of localization and user satisfaction initiatives. To manage data and efficiently ensure that different measures of Ethiopian organizations have access to passable information resources leftovers a major cultural issue^7^.

The current database used by the chosen enterprise POESSA—Private Organizations' Employees Social Security Agency for the purpose of testing the system quality, users’ satisfaction, and continuance intension is known as Amharic Extension Module (abbreviated as “AEM”), which was developed using PostgreSQL and integrated into open source databases.

The new type of localization style known by AEM has been installed and appeared in the context of information localization development. The development of AEM has been an unstoppable trend, driven by the advancement of information localization technologies and the support of governments^8^. AEM can meaningfully improve efficiency, resource utilizations, minimize cultural localization costs, and improve user overall cultural data retrieval by changing traditional localization models^9,10^.

Digital technology is increasingly contributing to the extent of cultural localization services, facilities and the expansion of cultures. Localization technology, in particular, is playing an important part in the supply of cultural facilities and services, owing to its ease of use, broad attention, and high productivity^11,12^.

AEM has the potential to alter and even bring back old-style data retrieval models, aptitude cultural improvement models, and systematic research models. Furthermore, through AEM, users can achieve virtual contact/communication with their boss to confirm appropriate change of information misused for report effects enhancement. As a result, the development and integration of AEM can decrease unwanted of information properties and payments by dealing with about of the external users who do not want to seek/find relevant information by reducing the cost of time of going to the organization they require. The inadequate good cultural resources may be bringing back for those in need taking to in such a way.

Programs and databases are typically culturally impartial, consisting of interactions among many mechanisms that are organized in precise designs and achieve part/all tasks. Using a shared language facilitates/services interactions among programmers, designers, maintainers, and users from all over the world. However, once we add morphological and social specificity to these databases/programs, many people feel more at ease with their natural language and would prefer to use it for day-to-day work as much as possible. Most current and existing cultural system environments include internationalization models, allowing developers to easily create software/apps that support multiple local languages^13^. Despite the fact that software includes internationalization models, the specific localization in some work done on Amharic local development has limitations that have a significant influence on managing Amharic local data in databases as well as other software systems. The minimum use and acceptance rate of AEM services/facilities by users with a large request/demand for databases expertise has hampered the development and widespread adoption of AEM in Ethiopia, despite the country's AEM market's rapid growth. The largest AEM company in Ethiopia, POESSA, has more than 15 thousand registered users, but only about 6.0% of those users are active^14^. Furthermore, according to Levy's^15^ research, localization and linguistic uses have a large removal rate, implying that request intension of users to continue using the application is lacking.

According to the preceding discussions, ordinary users' low approval and use rates have been a major impediment to AEM's long-term development.

Users with computer illiterates and elders may reject innovative systems in contrast to young people and people in their middle years because they lack the capacity to learn new things. However, even though there is a growing need for AEM services, digital cultural information systems worldwide face a significant obstacle: users' continued use of the systems. Continuous use is a key representative/typical of users' consistent, user performance and behavioral attitude that is also a crucial benchmark to determine as of the application/system has positive impact or not at all, according to research in the information systems field^16^. However, the current literature on AEM is mostly concerned with technological viability, cultural effects, early information system adoption, etc.

The behavioral intentions of users who have adopted AEM services are not given much consideration. As a result, there is still a research gap in this area that begs for additional theoretical and applied research. Only with more research focus on this area can AEM theories be improved upon and better marketing plans developed for AEM businesses. As a result, the combined ECTM with D&M ISS (abbreviation for Expectation-Confirmation Theory Model, and Deleon and Mclean Information System Success Model) model, the models with a good reputation in the area of information communication technology system intension of continuance and system quality, are incorporated into this study's AEM research. In fact, research from various fields has also supported the ECTM-D&M IS Success Model.

For instance, Kim et al.^17^ investigated database users' intention to continue using accommodations based on the ECTM. The findings indicated that the expectation confirmation has an important/significant impact on users' perceptions of the usefulness, helpfulness and satisfaction, with users’ satisfaction having the biggest impact on intension of users to continue using the product. Additionally, Cheng^18^ adopted ECTM to confirm the user's intent to continue with e-learning. The use of a sole/single classical model to clarify/explain the continued use of AEM by elderly people who are less computer literate is, however, limited. The ECTM-D&M ISS Model can clarify/explain the user's intention to continue using a product satisfactorily, but it overlooks practical factors that go afar satisfaction and confirmation. UTAUT (abbreviation of “Unified Theory of Acceptance and Usage of Technology”, on the other hand, excels at analyzing how users perceive technology and how it affects the environment. It can accurately gauge user behavior and adoption willingness. Research in a variety of fields, including implementing an information system modeling training^19^, virtual shopping^20^, and small pieces of lectures^21^, has established the validity of UTAUT. Although UTAUT is widely used by academics, this theory disregards the user's post-adoption behavioral intention. Many academics believe that in comparison to single models, comprehensive models can offer more worthwhile insights. This research supports this conclusion. The ECTM-D&M ISS Model and UTAUT are consequently combined in this study to adequately overcome the respective shortcomings of each model. Additionally, computer modelling such as density function theory (DFT) has an important role in the designing of the success models of Information Systems, and its calculations showed that the Amharic Language and its extensions have a fit for determination for the module we designed^22^.

According to Yaser et al.^23^, the broadly defined integrated model variables can influence a large percentage of localizations using Learning Management Systems. However, one of the integral factors in the expectation confirmation model (ECM), “Confirmation,” is discovered to be not that much important in the comprehensive integrated model. It was outlined the various important benchmarks throughout the LMS design phase as well as a strongly proposed optimum approach to a fully utilized localizations in the Learning Management Systems process at various institutions as well as higher education institutions. It was suggested that users are essential participants for whom the continued involvement tends to increase the interactions and exchange of knowledge.

Up on the given problems discussed, the research questions under the respective sub-chapter are posed in order to achieve the stated research objectives.

The main aim of this study is to propose an integrated model of ECTM, D&M ISS Model, and UTAUT to examine satisfaction of users and/or intension of continuance to use Amharic Extension Module.

As a result, the researcher has obtained the following questions to be answered at the conclusion of the research:

- What suggestions and opinions from users, how users satisfied on the system quality of the Amharic Extension Module implemented in the organizations?
- How can the integrated Amharic Extension module confirmation of users influence the satisfaction, perceived usefulness and continuance intension based on the ECTM?
- How performance expectation of the Integrated Amharic Extension module can influences the satisfaction of users based on UTUAT?
- How the system quality influences the confirmation of users upon the D&M ISS Model?
- Can UTUAT, ECTM and D&M ISS Model be successfully combined to form an integrated research model in the area of localization in open-source databases?

The findings and results of this study will be useful to the governmental and non-governmental organizations that used have local languages particularly the Amharic languages.

- For easiness of managing local data such as date/time, calendar, currency and others in specific to the Amharic languages and cultures;
- For reducing cost of time and cost of prices for users and organizations at large in the area of localization specific to Amharic cultures;
- For reducing costs for developers as they will be focus on handling Amharic languages and systems related instead of allocating with adaptations after developing the entire DBMS in English local, and hence the developed model is useful for further usability testing;
- The newly developed model is useful for understanding of Amharic language improvement in the digital world so that continual of user intension is better; and
- Use as an input for other works in the area of modeling in localization.

This study has a specific contribution for the department of Computer Information System and a general contribution in the area of Information Technology and Natural Language Processing in such a way that the department's developers and researchers are aimed and interested in the area of localization and model development for system quality, users’ satisfaction and continual user intension of a system. To say more on the contribution of this study, it provides on the acceptance and adoption of system quality for the confirmation of the performance expectations with respect to the users’ satisfaction and continuance intension in the area of Local Integration of open source databases and hence it out to inconsistent relevant conclusion in this area, and insufficient analyzing deciding factors in the area of local language integration in open source databases.

## Theoretical background

### Writing system in the Amharic language

Amharic, the official language of Ethiopia, is estimated to have more than 25 million native and second-language speakers^24^. The complete set of sounds/resonance for the Amharic local language is represented by a set of 39 phonetics, including eight vowels and 32 consonants^25^. The five basic types of consonants are stops, fricatives, nasals, liquids, and semivowels. There are also sounds that are similar to English sounds but are denoted by different symbols. Among symbols in the sound are [sx], [nx] and [ch]. The sounds [cx], [px], [tx], [xx], and [q] are Amharic characteristics but are absent from the English language^26^. All consonants in the Amharic language, with the exception of /h/ and /ax/, may be produced either through germination or non-germination, which is one of the language's most distinctive aspects of speech rhythm and also imparts a significant amount of semantic and syntactic functional weight^27^. The verb phrase follows the noun phrase in order of emergence. Sentences in Amharic can be divided into two categories: simple sentences and complex sentences, depending on how many expressions or phrases they contain. A complex sentence is made up of multiple noun and verb phrases as opposed to a simple sentence, which only contains one verb.

Therefore, after the integration of Amharic Extension Module into an open source database, it has been good to see the satisfaction of users and the continuity of users’ intension and the quality of the system so that such data management will be simple due to the variety and complexity characteristics of the Amharic language, culture, symbols, vowels, and calendars.

### Localizing scripts and calendars

The Arabic number system, the Ethiopian number system, or even words, locals and symbols from the Arabic numbers system can be used to represent numbers in the Amharic script system. Many religions and cultures all over the world have their own calendars that they use, which are not exactly the same as the Gregorian calendar used in the West. They still put up with it, though, according to the 12-month rule. 13 months make up an Ethiopian year, which is seven or eight years, late the Gregorian calendar. Because Ethiopians continued to use the similar calendar that the Roman church modified in 524 AD, they actually celebrated the start of the second millennium on September 11, 2007 (that was Meskerem 1, 2000 Ethiopian calendar). Except the last (13th) month known by the name Pagume that has five or six days depending on the leap year, the first 12 months have 30 days. Ethiopia uses its own calendar system, making it one of the few nations in the world to do so. Some significant holidays are observed in the nation on days that are different from those in the rest of the world^28^.

To enable both qualified and nonprofessional users who participate in website construction to do so comfortably, Lielet^29^ localized some of the contents of the open- source web construction and development tool (such as Joomla) into the locals like Amharic. The front and back end interfaces both provide Amharic translations. A digital keyboard is intended. A simulated keyboard is created and implemented to make Amharic locals/text entry simpler and the web texts/content more user-friendly. However, this project work also does not take into reflections/consideration the different Ethiopian locals of data types and symbols like currency, date/time format, calendar, format, regular expression, sorting/collation order, etc.

In their study from Sarfraz et al.^30^, the authors of the paper described how they localized a number of none open-source software programs created in English Language for Pakistani Urdu speakers. Web browsers, email clients, instant messaging clients, word processors, graphics editors, and tools for creating websites were chosen as the software programs. The requirement for localizing software was laid out in the paper as being that the software/application needed to be nationalized and internationalized. Since nationalized and internationalized development separates the resource files that need to be developed/customized (localized) for a given/target local, it enables an effective and suitable localization process.

According to Bader^31^, the analysis and methodology of translating websites from the source language (English) to the required/ target language (Arabic) are presented. Source files and database tables are the main factors the author took into account when localizing a website to the required/target language (Arabic). The source file for the target language was placed separately by the author after localization. Since the target/required language held by the localized/customized software requires database design adjustments during the software localization process. The author employs a standard technique for building a multilingual software/database, which is to make two or three tables.

Database tables are utilized differently for data storage and retrieval depending on the language and character set, claim^32^. As a result, MySQL has localization and internationalization models built in for adjusting to various local systems for effective data management. Different types/character sets for Oracle or SQL statements, languages related for error messages, and local time at various levels, such as column, table, database, the server, are all supported by MySQL.

As a result, as is evident from the reviews of such journals, it has been able to localize the Amharic scripts and calendars using a variety of localization strategies.

### The integrated Amharic extension module

Elmasri^33^ defined a database as a structured and organized collection of documents and data kept in a computer system. A program/system that accomplishes and executes the queries, as well as incorporates and integrates the data/information stored on the programs/system, would be part of a right great efficient and effective database system.

A suitable database is becoming increasingly necessary because of the enormously growing large size of local symbols or data that are a byproduct of modern life and require effective methods for managing and retrieval. Since converted data can occasionally cause information loss, it is crucial to store and access data in their original form^1^.

Open source databases have developed to the point where they can serve as stand-alone solutions for all enterprise data management requirements. Open source databases are becoming more and more popular, as per to reports from^34^, a virtual initiative to gather and publish information or data on DBMSs. Localization is the method of adjusting a feature to new language and cultural details of the content to a specific nearby aspect (locals). Ge'ez, a Semitic linguistics widely spoken in Eritrea and Ethiopia, is written using the Ethiopic script, which has more than 400 characters. The script/writings is still primarily used to characterize Eritrean and Ethiopian Semitic languages like Tigrinya, Tigré, and Amharic, even though the linguistic (Ge'ez) is only to be used in dialect speech as it only serves a language/liturgical function at this time^2,3^. Due to the use of Ethiopian calendar, a sizable amount of information/data in Ethiopic script must be stored in databases using the Ethiopian local system. The integrated and functional extension module for the Amharic locals for the successful management and storing and searching purpose has to be examined in its continuity as of users’ intension and satisfaction by making some sort of modality.

The trial ability compatibility of localization was found to be favorably correlated with the behavioral and character intention to use, according to the results. Li^35^ surveyed 303 Chinese users and discovered that trust, technology fear, and attitudes about utilizing mobile systems are all substantially correlated with intension of users' behavior to use Amharic Extension Module/AEM. The UTAUT model was expanded by^36^ for a structural and an empirical study of users' mobile system services and facilities in Taiwan. According to the findings, satisfaction is positively impacted by trust, conducive conditions, and performance expectancy. Also, the intention to use AEM for adoption is positively impacted by the database expertise and satisfaction.

The influence/impact of well recognized factors/parameters, such as customers' perceptions of service/facilities risk, quality, and surroundings, on the acceptance intention, on the other way, is supported by a large size of studies. In terms of supposed deal risk, Cocosila and Archer's summary of hazards affecting customers’ acceptance intention at the economic, emotional, and confidentiality levels is found in^37^. Later, Cocosila^38^ added two additional variables, period risk and communal risk, to further examine how these variables affected the intention to adopt. In order to determine how these effective quality parameters indirectly and directly affect acceptance intention through expectancy of performance,^39^ split localization and mobile application services/facilities at the quality level of system, quality of information, and quality of interaction.

In order to assess how the two specifically quality criteria affect users' continued intention to use localization applications through perceived utility, Chen^40^ divided quality factors into two categories: Database expertise’s service quality and local database users’ information quality. Moreover, resource accessibility and resource kind as contextual factors impacting users' propensity to use AEM from resource competition perspectives. In order to find indication for the influence of these environmental elements on people's acceptance of localization wearable devices, other researchers have proposed environmentally friendly factors, such as judicial protection, as a features of the website implementation for business^41^. All of the aforementioned research's findings have offered several angles for explaining why people utilize mobile medical technology. Yet, there is currently a lack of study on behavioral intentions after adoption.

While this is going on, governmental organization settings, samples, and infrastructure are inconsistent, which leads to conflicting applicable findings. In order to investigate the decisive elements impacting AEM usage intention, this study/research continues from the perspective of users with computer literates.

### PostgreSQL database localization

Several local systems and software supported by open-source databases such as PostgreSQL systems for effectively handling local information/data were given by the PostgreSQL Global Development Group^42^. PostgreSQL utilizes local features of the host operating system to manage various cultural and linguistic conventions (local data). A database cluster is automatically configured with local support when it is built with initdb. By default, Initdb will start the database cluster using the operating system's local features (execution environment), providing features such localized calendars, messages, and number formatting. By specifying the -local option, you can tell initdb specifically which local to use if you want to use a different local. PostgreSQL supports a number of local languages, including English (US), German, Swedish, French Canadian, etc.

### Localizations of MySQL database and Ethiopian locals

A research from^32^ presents that the different localities required the distinct approaches to manage a lot of applications. Therefore, MySQL databases included model of nationalization and internationalization and limitation for adjusting diverse social and phonetic shows for productive information the board. MySQL provides support for a variety of character sets for SQL statements, languages for error messages, and various levels of local time. For instance, at the level of column, table, databases, and the server, the procedure for addition of a new local to MySQL databases depends on whether it is simple or complex.

As of the reports of Clear IT Solution^43^, the Ethiopian language localization project includes features/modules that support Amharic localization for different customizations by expanding the base model's functionality. According to Rufael and Fekade^44^, a language pack for the open source database CMS is designed and developed as part of the localization process to transform the back-end and front-end interfaces of the web development tool such as Joomla. The language as a package in this development can be easily imported into Joomla and changed to a different language by users. Additionally, Joomla's virtual keyboard module allows users to encode Amharic text. Users can create a Joomla specific language pack for Tigrigna, Oromiffa and Amharic, using the translation component (translation memory). Despite the obvious advantages of translation memory systems, localization involves more than just translation. Language is not the focus of localization. It addresses additional cultural aspects and includes translation. However, system-level like Amharic locals such as date/time format, the calendar, regular expression, sorting/collection order, and others are not taken into account in this work.

### ECTM (expectation confirmation theory model)

Oliver^45^ first developed the ECTM (Expectation Confirmation Theory Model) as a modern and classical theory to investigate satisfaction of users and intension of continuity. Few researchers in the field of information communication technology systems began to incorporate ECTM into the study of information systems because of the positive explanatory power that ECTM demonstrated in traditional commerce. Bhattacherjee was the scholar who was most evocative. According to Bhattacherjee^16^, the behavior of information system users' repeated use is in line with the nature of consumer repurchase intention behavior, which compares the expected and actual effects of an invention or facilities or service to choose whether to have an additional purchase mechanism or reuse it. As a result, the information systems field can still benefit from the ECTM theory.

As a result, Bhattacherjee^16^ made the ECTM-D&M ISS Model theory for the system of information, which was basically based on the ECTM theory at large. The ECTM-D&M ISS Model theory was basically used to study intension of users to continue using a system because it relies on high forecast accurateness in the information system like fields. For instance, Lin^46^ used an empirical survey to determine that perceived usefulness, satisfaction and confirmation are the major factors that influence the intention of web portal and web site users. This study was based basically on the ECTM theory and examined the intension of users’ continuance on the web portals^47^. In order to investigate the issues of users' continued use of web portals and web sites included variables such as perceived confidentiality risk, supposed pleasure, perceived status, and public identification into the ECTM-ISS theory.

Gu et al.^48^ found that when the D&M ISS model and the ECTM theory were combined, the integrated model significantly outperformed the original ECTM in explaining Localization and mobile application users' intention to stay in the course. Additionally, a precise review of the relevant literature tells that the emergence of the ECTM and the ECTM-D&M ISS Model theory has established a solid and basic theoretical foundation for studies of users' continued consumption and use at large. However, this is not an implication that neither of these models is without flaws. Numerous researchers have altered the ECTM-D&M IS model and combined it with other impacting factors to examine/testing users' intent to continue using the system.

In order to make a better explanation on the users’ intension of continuance for AEM in both the database expertise and the ordinary users with computer literates, the study combines the ECTM-D&M ISS Model and UTAUT to sort the study on the research type of model more reliable and consistent and effective to the research theme/topic and subjects as well.

### UTUAT (“Unified Theory of Acceptance and Usage of Technology”)

Following the ECTM, the most influential and impacted theoretical model in the area and field of adoption of information systems and technologies has been UTAUT. The author in^49^ believed that the UTAUT model could serve as a standard for information technology adoption and an assessment instrument, explaining user acceptance and technology use by 65%. The research paper held that a solitary hypothetical model is fragmented in making sense of and foreseeing individual ways of behaving. Therefore, Venkatesh et al.'s summary of ECTM-related theories responded to concerns regarding “factors influence user cognition” by proposing the UTAUT, which stands for "Unified Theory of Acceptance and Use of Technology."

Among the many different theoretical models, a model combined and shared of ECTM and Information System Success models are associated with the adoption and application of information technology, together, these models form the UTAUT model. It is committed to investigating user adoption intentions as well as user behavior for new technologies and products. Performance Expectancy (PE) is the variable in the UTAUT model. Performance Expectancy (PE) has the power to directly influence user satisfaction and indirectly influence user intention.

In point of fact, the authoritative and traditional UTAUT has found numerous applications in the information industry. To examine intensions of users to use an innovative technology-driven product, Park et al.^50^ combined the UTAUT and the D& M ISS Model. Lu et al.^51^ utilized the UTAUT model to validate the mobiles shop intent of 656 Americans and 866 Chinese consumers. Oliveira et al.^52^ investigated Portugal users' intension of behavior and acceptance of mobile banking by integrating three models—the ITM, the UTAUT, and the TTF. The UTAUT model has been validated by various information systems fields, as can be seen. One type of information technology is localization, which is a platform for online text translation services.

The behavioral intentions of users with computer literates after adoption are the focus of this study. As a result, as the theoretical foundation for this study, we use the UTAUT model, which is regarded as authoritative and relatively traditional.

### D&M ISSs model

The D&M ISS Model was published in 1992^53^ for the purpose on theoretical and empirical Information System researches. Over the course of the past ten years, IS's role has evolved and changed. In a similar vein, academic research into measuring IS effectiveness has advanced over the same time frame. The review of Information System success measurement was informed by a review of more than one hundred articles, as well as all articles published in IS Research, JMIS, and MIS Quarterly at large since 1993. Therefore, the aim of this paper was to update/inform the D&M ISS Model and assess its usefulness and system quality in light of the significant shifts in Information System practice, particularly the emergence and rapid expansion of online shopping and e-commerce.

The main and primary goal of the original paper^53^ was to guide future researchers and consolidate earlier research on Information Systems success into a more intelligible body of knowledge. A multidimensional, complete model of Information System success was proposed on the basis of the communication investigation in the older papers like^54^, and the information system “influence” theory of^55^. According Shannon^54^, the practical level of communications refers to the correctness and effectiveness of the information-producing communication system. The degree to which the information successfully conveys the intended meaning is called the semantic level. The receiver's reaction to the information is the level of efficiency.

In the D&M ISS Model, technical and practical success is measured by “systems quality”; Semantic success is also measured by information quality; the success of effectiveness is measured by “use, user satisfaction, individual impacts,” as well as “organizational impacts.” Both Shannon^54^ and Mason's extensions of^55^ appear to be as usable as when we assumed and adopted them years ago, despite the passage of time.

### The gap in the literature

Generally, the new likely methods to support local language management and the ultimate goal of a quality system in the above related works are users’ satisfaction in the area of localization and continuity of users’ intension. However, the local systems specifically the Amharic Extension module is not tested whether they are correctly implemented in the organizations or not; and the users’ satisfaction and continuity of users’ intension in the system is not examined. Therefore, how the developed and installed system is successfully delivered as users’ willingness? How the extension module is satisfactory for the users? How users’ intension for continuity is achieved? To solve and find a mechanism for this, we propose that the solution is implementing a new model that catch up the combination of ECTM, UTUAT, and D&M ISS Models.

Accordingly, as it can be seen from the reviews of such journals we can make a new model from the combination of the three models so that it can fulfill the following gaps.

Researchers find out systems at the high level of either system qualification or information qualification or interactive qualification; but they didn’t verified and approved how these quality and good factors and parameters indirectly or directly affect the satisfaction and acceptance intension via performance expectation and perceived usefulness with respect to the system quality, particularly in the area of local language integration of open-source databases.

Many researches show/provide different perspective in different area adoptions and acceptances but there is no research showing on the acceptance and adoption of system quality for the confirmation of the performance expectations with respect to the users’ satisfaction and continuance intension in the area of Local Integration of open source databases and hence it out to inconsistent relevant conclusion in this area, and insufficient analyzing deciding factors in the area of local language integration in open source databases.

## Methods

### Research model

Several research and project works regarding the Amharic and other languages localization in their characters, different system quality models as well as users’ satisfaction and continual intension, and creating new models using a combination of different models for a continuity of users’ intension on systems and software extension module in the area of open-source databases have been discussed in the previous chapters. This is providing basic consideration to the possible limitations, opportunities, current status, and standards of supporting different locals in a database environment and creating a new model for continuity of users’ intensions. It also showed the major difficulties and problems that are holding back design and improvement of new models for satisfaction of users and continual intension in Amharic language integrated extension module. By considering the insight and output of the literature review, a new model has been developed for system quality to be sure of the satisfaction and continuity of users’ intension. Structural Model has been provided for the test of the users’ satisfaction and continual intension for the given system to make sure that system quality is approved. AEM was developed and implemented to support the localization purpose for Amharic language in Ethiopian organizations such as POESSA. We chose the Ethiopian organizations because of the Amharic Language is broadly speaking in Ethiopia, and Information Systems of Ethiopian organizations are not tested on the users’ satisfaction and continual intension of systems, as well as the one of the author of this research is currently working in Ethiopia and he knew the problems existing very well. The availability of the AEM in Amharic language will help to make the possibility of operators for execution as well as it can push the importance of users’ satisfaction and users’ intension for the continuity of the system.

**Figure 1:**
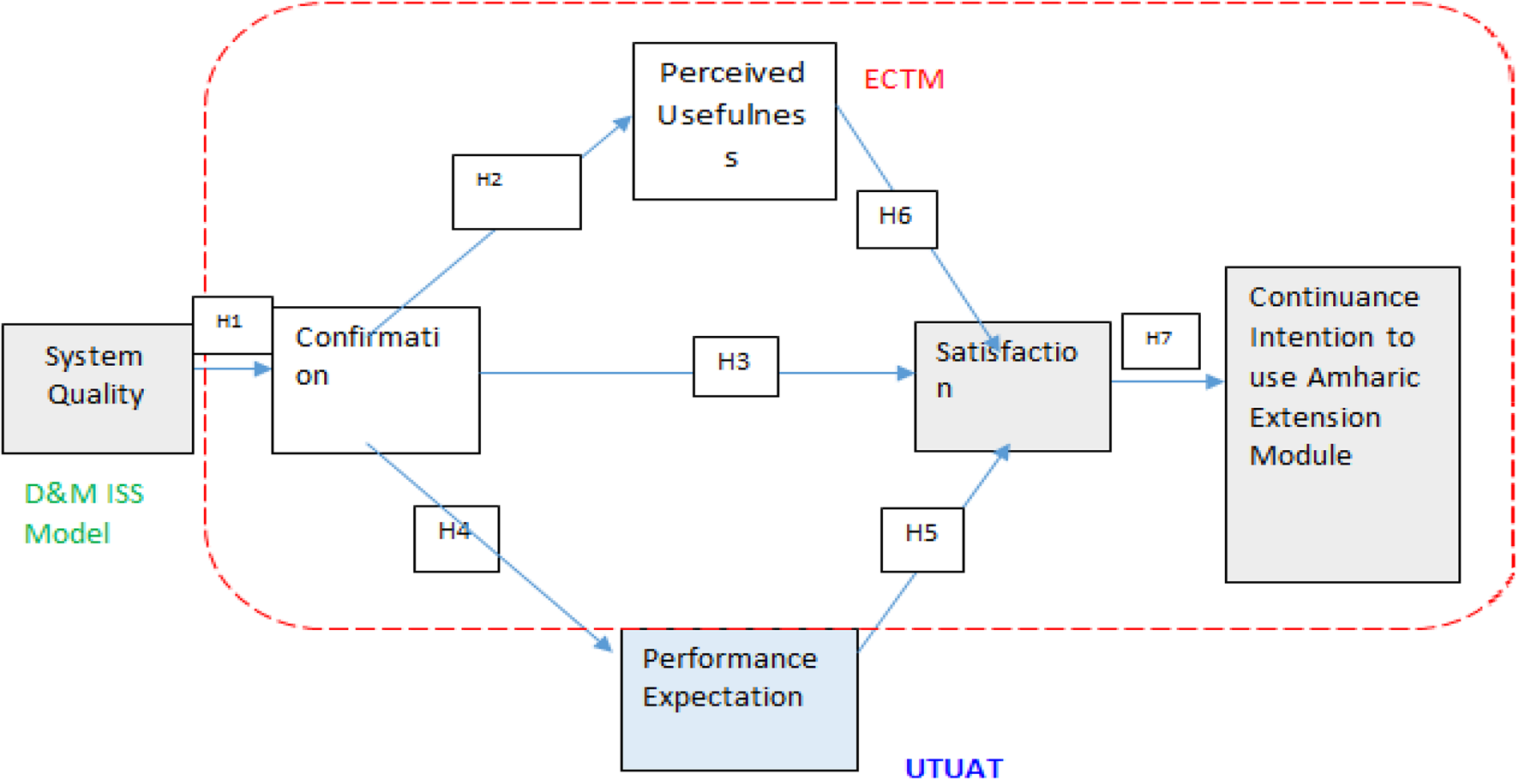
Model of the research.

The current body of research defines “continuance intention” as “behavioral willingness” following a first encounter with relevant services in the system, which can indicate how eager users are to continue using a particular IS in the near future. Gu et al.^56^ held the belief that protecting users' intent to continue using the system is actually more important than initial of use, which is the necessary step for the success and achievement of IS. This study develops a theoretical and practical model that is basically on the ECTM, UTUAT, and D&M ISS Model and can help advance the current AEM knowledge system in order to explain users' intention to continue using AEM, particularly when they are unfamiliar with database usage. Herewith, Fig. 1 demonstrates the research model.

### System quality on the confirmation of Amharic extension module

The confirmation of user expectations will be strengthened by improving the usability of the system or adding functions that are well integrated into professionals' workflow. According to Venkatesh et al.^49^ System Quality denotes to the extent to which a person's environment affects his confirmation of satisfactions and willingness to use an information system. Additionally, Dwivedi et al.^57^ discovered that users of systems tend to modify their attitudes in response to the knowledge and experiences of others.

According to Lu et al.^58^, System quality significantly affects users' intentions to adopt an information system. System quality was also mentioned as a significant influencing factor of users' adoption of any localization systems such as mobile technologies in open-source databases in^59^. Users using the system will inevitably be influenced by their friends and immediate bosses at office or other nearby individuals when using localization technologies. Database users will be more willing to use Amharic Extension module if others like their bosses support, encourage, or suggest it. This means that the intention of database users with computer literacy to continue using the system can be improved by others' favorable attitudes toward Amharic Extension module. Database experts with computer literacy may decide not to continue using Amharic Extension module if others like their friend and immediate bosses are against it or outright reject it. As a result, the following theories are put forth in response to the literature reviews:

#### H1

System quality has a positive influence/impact on confirmation of users’ expectations of the Amharic Extension module.

### Confirmation for perceived usefulness, performance expectation and satisfaction of users of Amharic extension module

According to Bhattacherjee^16^ the correlations between confirmation, performance expectation, satisfaction, continuance intension and perceived usefulness is stated and defined in the ECTM.

Confirmation and satisfaction, as well as satisfaction and perceived usefulness, can affect the continual intention using the system. In addition, satisfaction can be impacted by performance expectation. Many researchers looked at the connection between confirmation and satisfaction in various fields. The continuation intention of Korean mobile instant messaging users, for instance, can be significantly influenced by confirmation and satisfaction, according to Oghuma^60^.

The expectation confirmation can significantly impact users' perceptions of the satisfaction and usefulness of information sharing platforms upon the theory of ECTM^61^. According to the theory of ECTM, expectation confirmation can significantly impact how useful and satisfied users are with the information sharing platforms in the context of the sharing operational managements as well as sharing economy. The intention to continue using a system is also influenced indirectly by performance expectancy and satisfaction, according to Huang^62^. The following hypotheses are put forth in light of the literature review above:

#### H2

The confirmation of users’ expectation has a positive influence/impact on the perceived usefulness of the Amharic Extension module.

#### H3

The confirmation of users’ expectation has a positive impact/influence on the satisfaction of the Amharic Extension module.

#### H4

The confirmation of users’ expectation has a positive impact/influence on the performance expectation of the Amharic Extension module.

### Performance expectation for satisfaction of Amharic extension module

Performance expectancy concerns to perception of individuals and users intensions of how helpful a system is to his work. According to Riad et al.^63^, performance expectancy refers to users’ subjective opinions about how using a system will advance or improve their usage. The key to users adopting information technology is perceived usefulness. Database users with computer literates will be more likely to use Amharic Extension module if they believe it will increase the effectiveness of their day to day duties in data management. On the other hand, their user intention will be diminished if they believe that using the system won't benefit them in any way. Performance expectancy appears to be a major influence on users' intention to use the Amharic Extension module. Furthermore, Wu and Tian^64^ discovered that expectancy of performance can affect the most important influencing/impacting factors of continued use intention in addition to significantly influencing user satisfaction with the IS. The following hypotheses are created after the discussion above:

#### H5

The users’ performance expectation has a positive impact/influence on the users’ satisfaction of the Amharic Extension Module.

### Perceived usefulness on satisfaction of users

The degree to which users believe a system will improve their job performance is represented by perceived usefulness in ECTM^12^. AEM usage will determine his or her appreciation in a situation following adoption and will improve satisfaction. As a result, based on the ECTM, UTUAT, and D & M ISS models, we theorize and hypothesize the following item:

#### H6

Perceived usefulness has a positive influence/impact on users’ satisfaction of Amharic Extension module.

### Satisfaction of user intension on continuity of the Amharic extension module

According to Venkatesh et al.^49^, it is determined by how easily advanced database experts with high programming skills perceive Amharic Extension module to be used. The adoption of the localization and integration to open source databases by users may be significantly influenced by satisfaction, according to prior researches^59,65^. Database Administrators may experience concern when using Amharic Extension module due to highly experienced qualification and highly expectation factors. The database users with low experience of in computer systems will be happy with the Amharic Extension module and willing to keep using it if it is simple to use. On the other hand, if it has been extremely challenging to use, database experts will not be satisfied with the experience of users and users’ intention will be to stop using it. After such literature reviews, we theorized and hypothesized the following.

#### H7

The satisfaction of users has a positive influence/impact on the user intension on continuity of the Amharic Extension module.

### The research design

The study of this research work was conducted from September, 2022 to March, 2023, at POESSA (abbreviation for “Private Organizations’ Employee Social Security Agency”, Ethiopia, using the experimental and unified model-driven methods of research approach. The survey was carried out for all types of users who used computers as their day to day duties. The Amharic Local Extension Module (AEM) that has been developed and integrated to open-source database was used to model users’ satisfaction and continual intension in the research work. Measuring the intension and willingness of users with the business expertise to continue using the AEM, the data investigation and analysis of this study/research work is determined through Structural Equation Modelling, a method that associations statistics and qualitative and measurable hypothesis to measure and asses the cause-and-effect correlation.

### Participants

The researchers selected all employees that are using the AEM functionalities as ordinary users and database experts in the organization of POESSA with a total number of 385 copies of the questionnaires are distributed and of which 380 are returned as a valid responses, as Fraenkeal et al.^66^ suggested that “there are rules for determining the minimum size of groups to be calculated as 377” in such kind of researches. According to Kozmirchuk et al.^67^, the UTUAT and ECTM modeling are suitable to the localization purpose in any open-source databases particularly in the PostgreSQL server as an open source databases that was too implemented in the ISO and ANSI standards for the best services of backup and recovery. As Table 1 shows the demographic data of the research work samples shows, there are 152 females and 228 males taking up 40% and 60% of the total, respectively. When we see the ages of the employees, all the research work samples are aged between 18 and 55 years old. When we consider educational experience and background, those advancing from colleges/universities in the first degree are in the highest proportion, as high as around 51%. Even though most employees have many experiences, respondents using AEM has a few.

**Table 1:** Demographic sample characterization.

Variables		Numbers	Per (%)
Gender	Males	228	60
	Females	152	40
Age	18–25	61	16
	26–35	152	40
	36–45	110	29
	45–55	57	15
Education	High School or below Diploma First Degree Masters	49	13
		91	24
		194	51
		46	12
Experience	Yes	380	100

### Data collection tools

User satisfaction and users’ intension for continuity was conducted to test the combined models of UTUAT, ECTM and D&M ISS Model for Amharic Extension Module which was done based on structural equation modeling to validate its quality^68^. Researchers proposed a new model for evaluating a system quality, users’ intension for continuity, and users’ satisfaction of the developed, integrated and implemented system. Thus, the researchers created a new unified model to test the users’ satisfaction and intension of continuity. The new model and the hypothesis developed is tested and offered with respect to the development model of the Amharic extension module system. Then users’ feeling was taken from the questionnaires distributed. In the final of all these assessments and testing, the authors presented some sort of techniques for the usage and quality of the integrated extension module consequently based on assessment and testing of the model results. With this regard, this study engaged on quantitative data, which were collected over the questionnaire named “Users’ Feelings about their Satisfaction and Intension of Continuity about the Integrated Amharic Local Extension Module” that was installed and used by users of POESSA. The questions and items are of the questionnaires are taken on and adopted from^69–71^. The validity, quality, content, and legitimacy of the adopted and developed questionnaires were approved and checked by different professionals from different disciplines so that the questions are adequate to be used in this research. Using Cronbach’s alpha, 0.91 were determined for the reliability and consistency of the data collection tools, which is better than the actual value of 0.72^72^ that indicates a higher degree of validity. The prepared questions in the questionnaire comprise six parameters namely System Quality, Perceived Usefulness, Performance Expectation, Confirmation, Satisfaction and Continuance Intension from the three dimensions namely UTUAT, ECTM, and D&M ISS models. We take the System Quality parameter from the D&M ISS model; The Performance Expectation is taken from the UTUAT model; finally, all the remaining four parameters such as Perceived Usefulness, Confirmation, Satisfaction and Continuance Intension are taken from the ECTM. Accordingly, we obtain seven path hypothesizes and 21 items from all parameters. Actually, we have used a 5-point Likert scales that is to be determined as Strongly Disagree-SD (1), Disagree-DA (2), Less Agree-LA (3), Agree-A (4) and Strongly Agree-SA (5) for the replies of the questions and items because it is easy to interpret and understand. Strongly agree taken a positive attitude in the satisfaction and intension of continuity of the users/participants to the AEM/ Amharic Local Extension module.

### Methods of data analysis

The research used/conducted statistical method of data analysis like descriptive method of analysis (such as mean, average, correlation, and standard deviation), structural equation modelling, and measurement modeling to interpret/analyze the data obtained/gained by the questionnaire. SPSS and AMOS (an added SPSS module, used for confirmatory factor analysis, path analysis and SEM/ structural Equation Modeling) are the statistical packages that we used for the interpretation/analysis process.

### Procedure

It has been known that the Amharic Extension Module was developed, integrated and implemented into the open source database (PostgreSQL) in the high definition server with best quality in the chosen organization (POESSA). We started by introducing ourselves and needing the questionnaires to be filled by the volunteer participants who are using the Amharic Extension Module just for the purpose of academic studies. In order to make sure that participants are satisfied and they are willing to use in the future for the Amharic Extension module, the questionnaires are prepared to fulfill all the requirements of the users. After collecting the questionnaires, we tested the model we designed upon the given parameters and dimensions in the satisfaction and intension of continuity of Amharic Extension model. The first testing is testing on the model we designed. The next is testing the hypothesis developed. It has been assessed that a detailed and completed description about the Amharic extension module and the way how to fill the questionnaire has been given to those who participate in the survey before conducting the filling process, as it helps them in having an insight to the purpose of the survey. The participants and users were asked if they have been used for at least six months and above. The participants were also asked to use and practice on the Amharic Extension module if they were not habituated before, for at least two weeks. Then after, participants/users of the system were provided and given with respective questionnaire. To select representative number of participants, we have used random sampling way of technique. The choice of participants and users in the survey process was taken by considering their practical usage on the AEM and knowledge of the basics of computer skills and positions in the organization they have had. Finally, just after getting all responses from respondents and participants through the questionnaire, we calculated and measure each dimension of the parameters of the questionnaire.

*All methods were carried out in accordance with relevant guidelines and regulations. All experimental protocols were approved by the organization POESSA.

### Informed consent.

Informed consent was obtained from all subjects and/or their legal guardian(s).

## Evaluation results

### Measurement of the new model

We started by performing a convergent validity and reliability analysis. The questionnaire's reliability analysis was done using SPSS. Every variable has a Cronbach's alpha of 0.91 that is higher than the average level of 0.71. This shows that the measuring model is reliable (Hair et al. 2010). The questionnaire's convergent validity was tested using AMOS, an additional SPSS module used for confirmatory component analysis, path analysis, and SEM/structural Equation Modeling. The CR—“composite reliability” of each variable is larger than 0.8, and all loadings are larger than 0.71. The AVE—“average variance extracted” of each variable, however, is more than 0.55, supporting the measurement model's validity. The SIL—“standardized item loadings”, CR—“composite reliability”, AVR-average variance retrieved, and Cronbach’s Alpha values are shown in the Table 2. The majority of standardized item loadings are more than 0.71, as shown in the Table 2 as well. Every AVE and CR exceeds 0.51 and 0.81, respectively. Moreover, every Cronbach Alpha value exceeds 0.81. This demonstrated the very convergent validity and reliability of the study^73^.

**Table 2:** SL, average, alpha and CR.

Dimensions/models	Parameters/factors	Item num/ques num	SIL	Alpha	Average variance	Comp. rel
D&M IS success model	System quality (Par-1)	1	0.811	0.821	0.632	0.819
		2	0.831			
		3	0.809			
UTUAT	Performance Expectation (Par-2)	4	0.761	0.871	0.651	0.837
		5	0.724			
		6	0.719			
		7	0.791			
		8	0.775			
		9	0.742			
		10	0.711			
ECTM	Perceived usefulness (Par-3)	11	0.880	0.832	0.593	0.823
		12	0.793			
		13	0.821			
	Confirmation (Par-4)	14	0.792	0.835	0.606	0.853
		15	0.771			
	Satisfaction (Par-5)	16	0.892	0.832	0.584	0.851
		17	0.821			
		18	0.799			
	Continuance intension (Par-6)	19	0.782	0.809	0.574	0.801
		20	0.801			
		21	0.821			

It is possible to assess the variables by contrasting the AVE “square root and correlation coefficients” across variables, perception validity—which is defined as the little correlation and significant alteration between hidden variables. A variable's perception validity is good if its correlation coefficient with another variable is greater than the average variance variable’s square root^73^. The “correlation coefficients” between the six latent variables are listed in Table 3. Each variable’s AVE square root is represented by the value on the diagonal line (shown in bold numbers in the table).

**Table 3:** Correlation and validity matrices.

	Var-1	Var-2	Var-3	Var-4	Var-5	Var-6
Var-1	0.782					
Var-2	0.329	0.793				
Var-3	0.288	0.367	0.765			
Var-4	0.561	0.329	0.491	0.721		
Var-5	0.445	0.391	0.421	0.601	0.743	
Var-6	0.512	0.433	0.293	0.492	0.652	0.719

Each variable's square root of AVE ranges from 0.719 to 0.793. The correlation efficiency between several variables has an absolute value that is less than 0.7. The “correlation coefficient” between a variable and other variables is obviously lower than the square root of the AVE of each variable. This shows that the six latent variables have favorable discriminant validity. The factor “correlation coefficients” and the “square root” of AVE are shown at the diagonal line of Table 3.

Furthermore, we examined the hetrotrait-monoterait ratio (HTMT), as described by Hessenler et al.^74^. Discriminant validity between two reflective constructs is demonstrated if the HTMT score is less than 0.75. The results in ratio of the HTMT against our data are displayed in Table 4. The threshold is met by all values.

**Table 4:** Hetrotrait-monoterait ratio (HTMT).

	Var-1	Var-2	Var-3	Var-4	Var-5	Var-6
Var-1						
Var-2	0.342					
Var-3	0.671	0.576				
Var-4	0.441	0.612	0.621			
Var-5	0.387	0.347	0.265	0.511		
Var-6	0.573	0.451	0.543	0.319	0.418	

### The structural model

The accepted “Structural equation modeling software” AMOS was used to evaluate the structural model. The “chi-square” (X2), the “degrees of freedom” (D.F), the “adjusted goodness-of-fit index” (AdGoFiIn), the “root mean square error of approximation” (RoMeSqErAp), the “incremental fit index” (InFiIn), the “goodness-of-fit index” (GoFiIn), “comparative fit index” (CoFiIn), and “normal fit index” (NoFiIn) were all used to evaluate the model fit and all had improved actual values than the suggested values as shown in Table 5. This proposes that the structural model is a noble fit for the data.

**Table 5:** The structural models’ fit indicators.

ModelFitIndices	X2/D.F	AdGoFiIn	RoMeSqErAp	InFiIn	GoFiIn	CoFiIn	NoFiIn
RecommendedValue	2–4	> 0.81	> 0.052	> 0.911	0.912	0.900	0.921
ActualValue	2.91	0.993	0.063	0.971	0.923	0.951	0.943

**Figure 2:**
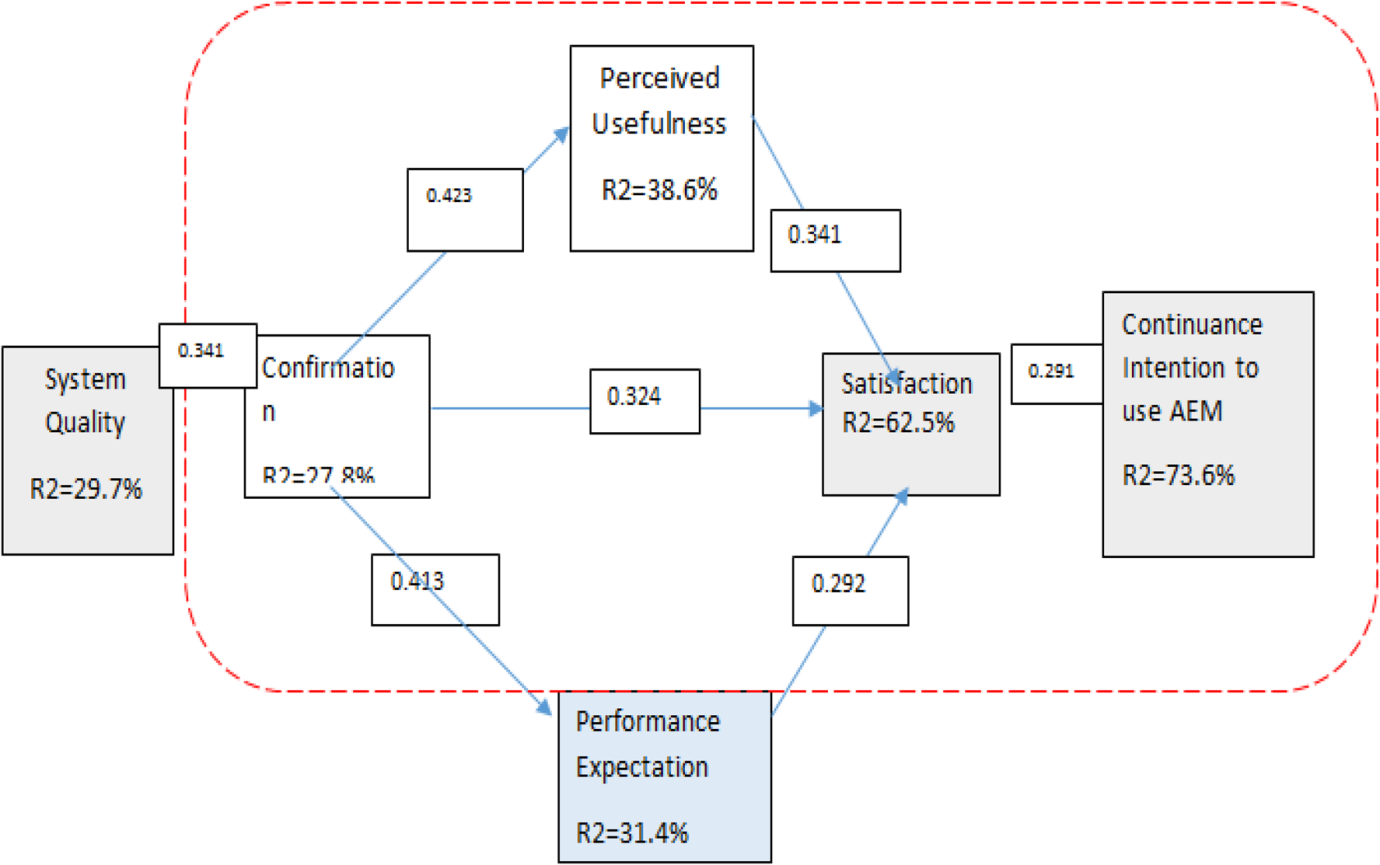
Structural model results.

The results of the AMOS estimation are presented and displayed in Fig. 2 and Table 6. The model accounts for 73.6% of the intention to keep using AEM. The continuation intention H7-related hypothesis is confirmed. The model supports the hypothesis and accounts for 31.4% of the difference in performance expectancy (H4). This model validates the relationships between the drivers of satisfaction, performance expectancy, perceived usefulness, and confirmation, and it accounts for 62.5% of the variation in satisfaction (H3, H5, H6). 38.69% of the difference in perceived usefulness can be explained by this model. The findings also support the theories linking perceived usefulness and confirmation (H2), and confirmation to system quality (H1). The next paragraphs provide a summary of the analysis' findings.

**Table 6:** Hypothesis TEST results.

		Estimates	S.E	*P* value (C.R)
H1	System quality -> confirmation	0.341	0.029	(13.327)***
H2	Confirmation -> perceived usefulness	0.423	0.039	(10.812)***
H3	Confirmation -> satisfaction	0.324	0.032	(8.431)***
H4	Confirmation -> performance expectation	0.413	0.027	(12.638)***
H5	Performance expectation -> satisfaction	0.292	0.033	(9.274)***
H6	Perceived usefulness -> satisfaction	0.341	0.037	(11.831)***
H7	Satisfaction—> continuance intension	0.291	0.024	(7.921)***

In explaining the confirmation, the quality of system was statistically important (β = 0.341; *p* < 0.01), supporting Hypothesis H1. According to the findings, the construct that best explained system quality in AEM was confirmation. That is to say: ceteris paribus, when confirmation increased by one standard unit, system quality improved by 0.341 standard units. 29.7% of the difference in system quality is explained by the model.

Hypothesis H2 as well as H4 were supported by the perception of usefulness and performance expectation, which were statistically important in clarifying the confirmation (β = 0.423; *p* < 0.01) and (β = 0.413; *p* < 0.01), respectively. The findings show that confirmation was the key concept in illuminating the perceived usefulness and performance expectation of the Amharic Extension Module/AEM. In another ways, ceteris paribus, when confirmation improved by one standard unit, perceived usefulness also improved by 0.423 standard unit and performance expectation also increased by 0.413. 38.6% of the difference in perceived usefulness is explained by the model, and 31.4% of the difference in performance expectation is explained by the model.

Confirming Hypotheses H3, H5, and H6, the confirmation (β = 0.324; *p* < 0.01), expectancy of performance (β = 0.292, *p* < 0.01), and perceived usefulness (β = 0.341; *p* < 0.01), respectively, were statistically important in clarifying satisfaction. Given that satisfaction improved 0.341 standardized units, for every one standard unit that perceived usefulness increased, the findings suggest that usefulness was the most significant hypothesis to explain satisfaction. The model accounts for 62.5% of the variance in AEM satisfaction.

In explaining continuing intention, the satisfaction (β = 0.291; *p* 0.01) was statistically important, that is supporting Hypotheses H7. Given that continuance intention increased by 0.292 standardized units, when satisfaction increased by one standardized unit, the findings suggest that satisfaction was the most significant hypothesis to explain intension of continuance. 73.6% of the variation in the intension of continuance of AEM is explained by the model.

## Discussion

This chapter presents the overall discussions of results of the research work. The Amharic extension module defines the necessary operations for Ethiopian numbers, Ethiopian currency, and the Ethiopian calendar, so you can manipulate them freely. To manage geez numbers with the Amharic extension we can use the integral data type and the to_number() function. This is the reason that existing systems of the databases are assuming geez numbers as separate characters^75^. Managing the Amharic language’s local data in the existing Western and Gregorian pattern convention DBMS resulted in information loss. For example, if you insert a value into the some columns, the amount is stored in different characters. The Western existing DBMS cannot perform the operations required to work with Amharic local data and characters. However, the Amharic extension module allows various operations on local Amharic data according to local conventions. Amharic local data is managed in an existing DBMS^76^, but using of such applications should be evaluated its users’ satisfaction and intension of continuity by developing integrated models. From the promising and encouraging test of the study, it is clear that users of POSSA have no problem to manage the data in the extension module. The validity of the system quality whether it has been correctly implemented or not is approved based on^69^. The survey also considers the system’s reliability, functionality, efficiency, maintainability and usability from the participants’ opinions for the system quality.

On the other hand, examining the variables affecting users with computer literate who continue to use Amharic Extension Module/AEM is the goal of this study. The ECTM-D&M ISS and UTAUT are used as the foundation for the creation of an integrated model. Also, the structural equation model is used to assess the seven assumptions that were previously mentioned. The seven hypotheses posed by this research are all supported, according to the analysis results.

At the beginning, system quality can have a considerable impact on confirmation of users (β = 0.341; *p* < 0.01). This can lead improving the system quality of the application AEM is confirmed for improvements. Then after, confirmation can have a considerable impact on perceived usefulness (β = 0.423; *p* < 0.01); Satisfaction (β = 0.324; *p* < 0.01); and Performance Expectancy (β = 0.413; *p* 0.01). These findings are in line with earlier research/study findings^58,60^. Confirmation is when you compare how satisfied people are before and after using a technology. Users with the computer literate may be satisfied with their initial as well as later use of AEM application, which can lead to improved performance expectancy, perceived usefulness and user satisfaction.

In addition, satisfaction of users (β = 0.291; *p* < 0.01) could meaningfully impact/influence continuation intentions. Satisfaction, on the other way, is influenced meaningfully by confirmation (β = 0.324; *p* < 0.01), perceived usefulness ((β = 0.341; *p* < 0.01) and performance expectation (β = 0.292; *p* < 0.01). Of these, the impact of perceived usefulness on going concern is most important. The results suggest that the more AEM helps improve the efficiency of users’ satisfaction on localization and improve the computer literation for both young and elder users, the happier and more willing they are to continue AEM. This is because AEM is superior to traditional cultures by own languages delivery models, and if the younger database expertise can improve their localization performance more conveniently, they will be more likely to continue their AEM. Suffice it to say that they may have strong intentions to use it and has more satisfactions. The results and findings of this model are consistent with previous and earlier studies illustrated in^48,64^.

Finally, satisfaction of users (β = 0.291; *p* < 0.01) influences significantly continuation intentions. This result is consistent with previous studies and the original ECTM hypothesis^77,78^. Because both database expertise and ordinary users of databases live in such localization environments, the satisfaction of users’ impact on the use of database applications and their localization extensions. Currently, the system applications usage status of the majority of the employees is optimistic. If these groups were able to encourage other employees to use the AEM in managing their data of day to day duties, then all users of the databases would demonstrate positive attitudes towards the Amharic Extension Module and continue to use the AEM. Meanwhile, the complete infrastructure such as broad band Internet, WiFi and Intranet can also affect to use the system for those users’ willingness to continue with the AEM.

Generally, the full value of Amharic Extension Module can only be realized with long-term use by database users with computer literate. Previous and earlier research has focused on the functional and non-functional design and benefits of localization and other systems and applications. In contrast, this study begins by evaluating the system quality, and then examining the factors that influence satisfaction of users and the intention to continue Amharic Extension module from the perspective of users with computer literates. The researcher validates the efficiency of the combined ECTM-D&M ISS and UTAUT model with the Amharic Extension module/AEM. This may provide new and important evidence for other researchers. We have suggested that the continuance intension of the users’ satisfaction and acceptance of AEM based on these three theories. In addition, the findings of this survey may have some impact on management. Based on the influencing factors, this study proposes measures to further improve AEM, increase the willingness to continue AEM among the young and elder users of the system, and further promote the value of Amharic Extension module/ AEM among users of the system.

From a theoretical point of view, ECTM-D&M ISS and UTAUT are comparatively imposing traditional theories in the area of IS and has been widely validated by many researchers. However, each model in their own has limitations. ECTM can proactively explain the user's intention to continue, but ignores external factors that influence user expectations and the quality of the system. UTAUT is well suited for studying the psychological characteristics and attitudes of users. D&M ISS is well only suited for system quality as in terms of people confirmations. Both UTUAT and D&M ISS are good at measuring the user's initial acceptance intent and system quality, but ignore the user's behavioral intent after using it. In order to better clarify system quality, users’ satisfaction, users' continuation intentions and increase consistency with research models and research themes, in this study the integrated ECTM-D&M ISS and the UTAUT to assess the Amharic Extension Module users’ satisfaction and continuation intention. Compared to or UTAUT, ECTM, or D&M ISS alone, the integrated model can deliver more explanation for satisfaction and continuing intent. The consolidated model can clarify 73.6% of the going concern variation. This is significantly greater than the constructed original ECTM of 40.5%. It facilitates the development of continuing research and is even of great academic importance for the introduction of information systems. In the future, it can combine these three types of opinions to investigate the user's acceptability of other information systems in the domain. We believe that integrated models can provide more insight than any single research model or perspective in the continuity of users’ intension.

## Conclusion, implications and future works

### Conclusion

The structural and measurement investigational results of this research work approve that, since the means of users’ opinions were very high, the Amharic Extension module is well designed, integrated and implemented technically as of a test of its evaluation. As an alternative plan to have a simple translation of local systems, Amharic Extension Module is gaining attention from the governmental and non-governmental organizations in Ethiopia to individuals. However, there is still no research approach to comprehensively assess Amharic Extension Module system quality, users’ satisfaction, and continuation intentions from the perspective of users with computer literates. To fill the literatures in the research gap, we have combined and integrated three influential theories, namely D&M ISS, ECTM and UTAUT, to create an original and new research models. We collected data from POESSA in Ethiopia respondents and tested and evaluated an integrated survey model to identify key going concern precedents. This will effectively fill a gap in localization research for uses’ satisfaction and continuance intension of systems. It was also the first time that a quality of system is evaluated and users’ satisfaction and user intentions were tested in computer literates in the organization employees’. Our findings suggest that the integrated research model has favorable explanatory power in the localization of languages.

This may help demonstrate the validity of system quality research models when analyzing user satisfaction and intentions for continued Amharic Extension module use in these users. Outcomes indicate that system quality can influence on the confirmation of a system, and confirmation can influence perceived usefulness, satisfaction, and expectations of performance, and again the satisfaction of users directly influence Amharic Extension Module continuation intentions. This study can provide a solid foundation for improving the ongoing concern models that are separately implemented. On the other hand, the results of this survey are also important for the Amharic Extension Module expertise/ developers to understand user expectations and perceived usefulness for the design, improvement and implementation of Amharic Extension module services in any organization. All these implemented and integrated services and features of the Amharic Extension Module can initiate and stimulate users’ willingness to continue Amharic Extension module and desire for long-term localization managements.

As on-premises data continues to grow in many areas and fields, how to efficiently succeed that data and information based on the users' on-premises needs becomes important. Most data and information management programs are developed and implemented to support some specific cultures. Apart from all the government and non-government efforts, individuals and separable developers and designers also need to be motivated to participate in the localized data and information management development process, system quality, and users’ satisfaction and continuance intension. In this research, we took a first phase towards the important or ultimate goal of attaining full multi-local functionality in system quality, users’ satisfaction and intension for continuity in an open source database systems.

This research will help Amharic localizations and particularly on extension modules in open source databases providers develop effective strategies to improve user demand and user engagement. First of all, system quality can have an important impact on confirmation; and confirmation can have an important impact on satisfaction, performance expectations, and perceived usefulness. Performance expectation and perceived usefulness can directly and significantly influence on satisfaction. The users’ satisfaction can further directly and significantly impact and influence continuation intentions, and can indirectly impact/influence continuation intentions through perceived usefulness and performance expectancy. Of these, the direct impact of performance expectations on going concern is most important. Therefore, when developing Amharic Extension Module capabilities, localization application providers should consider the expectations of users’ with low computer literates in relation to Amharic local extension capabilities or services. Therefore, Amharic Extension Module not only provides detailed manual instructions to reduce the time it takes for seniors to get used to using AEM, but also provides timely instructions if juniors encounter problems using AEM. It is suggested that AEM developers should focus on its practicality, preserve a close relationship with its users, and be directly aware of their prospects and requirements for localization of data management and systems for the users.

Moreover, they should fairly acknowledge the applied powers and boundaries of the Amharic Extension Module and avoid exaggerating the advertised capabilities of localization. This affects the user's willingness to continue.

### Theoretical implications of the model

The theoretical implication of this model suggests that the actual use of technology is determined by behavioral intention. The perceived likelihood of adopting the technology is dependent on the direct effect of the key constructs, namely performance expectancy, effort expectancy, social influence, and facilitating conditions. Therefore, from a theoretical point of view, ECTM-D&M ISS and UTAUT are comparatively imposing traditional theories in the area of IS and has been widely validated by many researchers. The broadly defined integrated model variables can influence a large percentage of localizations using Learning Management Systems. However, one of the integral factors in the Expectation Confirmation Model (ECM), “Confirmation,” is discovered to be quite important in the comprehensive integrated model. It was suggested that users are essential participants for whom the continued involvement tends to increase the interactions and exchange of knowledge.

### Practical implications of the model

The practical implication of this model, on the other hand, suggests that the actual use of technology is determined by the continual intention of the system by the users. From a practical point of view, the study should also invite seniors to make product improvement suggestions so that the Amharic Extension Module can effectively improve their day today activities performance for all governmental and non-governmental organizations in the Ethiopian context. In addition, AEM should take no power to improve the users’ and participants’ understanding of the Amharic Extension module’s reasonable advantages over other localization IS in function. Therefore, the emphasis would be on emphasizing the situations that enable the design of information systems. System interfaces should be brief. Text, images, special characters, and videos should be properly coordinated and spread to avoid data overload, and system quality should be enhanced.

Therefore, it can increase awareness and promotion of Amharic Extension Module itself. Meanwhile, take advantage of the multi-channel socialization platform to launch various topics and activities on a regular basis to invite users' attention, improve user interaction, and reduce the space between users and operators. In addition, Amharic Extension Module has the potential to collaborate with internet celebrities and starts to develop the functionalities of sorts. These thought leaders can facilitate and strengthen the willingness and intension of the users’ to stay with the Amharic Extension Module. Additionally, supportive conditions can have an important impact on users’ willingness/intension to continue.

### Limitations and future works

This study turns around intent to continue Amharic Extension Module/AEM in database users with computer literates. Despite sufficient dependability and validity attained by the hypothetical model constructed under such research work, this study cannot circumvent some limitations due to inadequate manpower and measurable resources. Effectiveness of System quality, users’ satisfaction of the system and Intention to continue in the users of the Amharic local Extension module is explained from the combined perspective of ECTM-D&M ISS and the UTAUT.

Future investigators can consider more influential factors and variables such as supposed value, belief and confidentiality to further advance the scientifically improved and possibility of research models. The other limitation of this research work is all respondents to this survey are only from one organization i.e. POESSA. Our findings are not considered other organizations that need localization of Amharic language. Therefore, the feasibility of the research model in other governmental and non-governmental organizations should be further validated. After all, all the respondents were users of databases in the POESSA with computer literate. In the future, all employees of any organization that used to have Amharic localization may also be included to examine differences in users’ satisfaction and continuing intentions between users within and out of the organization.

## Data Availability

The datasets generated and/or analyzed during the current study are not publicly available due to the in permission of the respondents but are available from the corresponding author on reasonable request.
